# Crowdfunding for Complementary and Alternative Cancer Treatments in Tijuana, Mexico: Content Analysis

**DOI:** 10.2196/52018

**Published:** 2024-08-14

**Authors:** Jeremy Snyder, Marco Zenone, Ashmita Grewal, Timothy Caulfield

**Affiliations:** 1 Faculty of Health Sciences Simon Fraser University Burnaby, BC Canada; 2 Faculty of Public Health and Policy London School of Hygiene and Tropical Medicine London United Kingdom; 3 Faculty of Law University of Alberta Edmonton, AB Canada

**Keywords:** cancer, crowdfunding, Tijuana, CAM, patient, patients, insurance, crowdfunding platforms, GoFundMe, GiveSendGo, cancer clinic, Mexico, campaigns, cancer treatment, medical intervention, CAM cancer treatments, misinformation, alternate care, women's health, internet research, international medical tourism, alternative cancer therapy, financial toxicity

## Abstract

**Background:**

Complementary and alternative (CAM) cancer treatment is often expensive and not covered by insurance. As a result, many people turn to crowdfunding to access this treatment.

**Objective:**

The aim of this study is to identify the rationales of patients with cancer seeking CAM treatment abroad by looking specifically at crowdfunding campaigns to support CAM cancer treatment in Tijuana, Mexico.

**Methods:**

We scraped the GoFundMe.com and GiveSendGo.com crowdfunding platforms for campaigns referencing CAM cancer clinics in Tijuana, initiated between January 1, 2022, and February 28, 2023. The authors created a coding framework to identify rationales for seeking CAM treatment in Tijuana. To supplement campaign metadata, we coded the beneficiary’s cancer stage, type, age, specific treatment sought, whether the beneficiary died, gender, and race.

**Results:**

Patients sought CAM cancer treatment in Tijuana because the (1) treatment offers the greatest efficacy (29.9%); (2) treatment offered domestically was not curative (23.2%); (3) the clinic treats the whole person, and addresses the spiritual dimension of the person (20.1%); (4) treatments are nontoxic, natural, or less invasive (18.2%); and (5) clinic offers the newest technology (8.5%). Campaigns raised US $5,275,268.37 and most campaign beneficiaries were women (69.7%) or White individuals (71.1%).

**Conclusions:**

These campaigns spread problematic misinformation about the likely efficacy of CAM treatments, funnel money and endorsements to CAM clinics in Tijuana, and leave many campaigners short of the money needed to pay for CAM treatments while costing beneficiaries and their loved one’s time, privacy, and dignity. This study affirms that Tijuana, Mexico, is a very popular destination for CAM cancer treatment.

## Introduction

Traveling abroad for complementary and alternative (CAM) cancer treatments—understood as medical interventions that are outside of standard medical care—is common in North America and Europe [1,2]. Motivations for seeking CAM cancer treatments include the belief that alternative care alone is curative, desire for control over one’s care, addressing the side effects of conventional treatment, attending to the needs of the whole person (including their emotional and spiritual well-being), and preservation of hope for better health [3-5]. For people seeking CAM cancer care abroad, private and public insurance typically does not reimburse any or all costs of this treatment. In these cases, crowdfunding can serve as a means of accessing CAM cancer care through helping the beneficiary not only to afford the treatment itself but also to pay for indirect expenses like travel, accommodation, and time off work [6].

Previous scholarship on crowdfunding for CAM cancer treatments has identified several concerns with this practice. These campaigns can spread misinformation about the safety and efficacy of CAM cancer treatments, potentially reaching large audiences through social media [7]. People with cancer who forgo conventional treatment in favor of alternative modalities may have poorer health outcomes [8]. Campaign beneficiaries are generally very ill and have a late-stage cancer diagnosis. The preservation of hope for a cure or extended life may come at financial costs and divert time from palliative care and other activities [9,10].

Prior analyses of crowdfunding campaigns for CAM cancer treatments have identified Mexico as a common destination. Peterson et al [7] found that 81.9% (N=194) of US-based campaigners on the GoFundMe crowdfunding platform who sought CAM cancer treatment abroad intended to travel to Mexico. Within Mexico, the Tijuana region on the US border is especially popular. The 5 most commonly named facilities in 1 study of crowdfunding for CAM cancer treatments were all located in Tijuana, Mexico [9]. These connections may be further reinforced by clinics in Tijuana encouraging potential clients to use crowdfunding to pay for their services [11].

The aim of this study is to build on this previous scholarship on crowdfunding for CAM cancer care by looking specifically at crowdfunding campaigns to support CAM cancer treatment in Tijuana, Mexico. Our aims in doing so are to revisit analyses of crowdfunding for CAM cancer treatments following the removal of COVID-19–related travel restrictions. We also seek to better understand the demographics of and rationales for people seeking CAM treatment in a specific, highly popular destination catering to patients from abroad.

## Methods

### Overview

We searched the GoFundMe and GiveSendGo crowdfunding platforms from March 1, 2023, to March 7, 2023. These 2 crowdfunding platforms were selected because GoFundMe is the largest host of health-related crowdfunding campaigns in North America, while GiveSendGo has emerged in North America as a home for Christian and politically conservative campaigners, often as an alternative to GoFundMe [12,13]. The search was conducted using the clinic’s name or the locations “Tijuana” or “Baja” with “cancer” and “alternative.” Provider names were compiled from publications on alternative cancer providers in the scholarly [9,14] and gray [15,16] literature. This list was expanded as additional facilities were identified during the review of resulting crowdfunding campaigns. The search was carried out using a database of scraped campaign data from both platforms and the platforms’ internal search engines. Campaigns initiated between January 1, 2022, and February 28, 2023, were then selected. This process identified 484 campaigns (GoFundMe n=432, GiveSendGo n=52).

The scraped data for these campaigns included the campaign URL, title, text, updates, funding requested, funding pledged, number of donations and online shares, creation date, and currency type. GoFundMe campaigns also included the campaigner’s city and country location. These campaigns were reviewed for inclusion as seeking funding to access CAM cancer treatment in Tijuana, Mexico. This process removed 124 campaigns, leaving 360 campaigns that met our inclusion criteria. One clinic, Hope4Cancer, also operates facilities in Cancun, Mexico. Campaigns for treatment at this clinic were included regardless of the intended location as the specific location was often unclear and they captured a similar practice. During this review, we confirmed the clinic name where possible and recorded information about the beneficiary’s cancer stage and type, the beneficiary’s age, the treatment cost, the treatment sought, and whether the beneficiary had died. Funding requested and pledged were converted to US dollars for non-US currencies using the exchange rate for the date the campaign was created.

We independently reviewed 10% of included campaigns with a focus on campaigners’ stated reasons for selecting Tijuana, Mexico, as a destination for CAM cancer care. Based on this review and after discussion among all authors, we identified five rationales for seeking alternative cancer care in Tijuana. (1) Treatment in Tijuana offers the greatest efficacy in terms of cancer treatment. Campaigners could support this rationale with appeals to specific and comparative success rates, patient testimonials positively discussing the efficacy of the treatment they received, and claims that these treatments improved success rates by supplementing treatment available domestically. (2) Treatment offered domestically was not curative. Campaigns with this rationale could include statements that the recipient was previously offered a poor prognosis, directed toward hospice or palliative care, or variations on the theme that trying some form of care was better than having no potentially curative care. (3) The facility in Tijuana is caring, treats the whole person, and addresses the spiritual dimension of the person. This rationale included depictions of specific, caring interactions with staff in Tijuana, references to the spiritual and religious convictions of staff in Tijuana, or references to a “whole person” approach. (4) Treatment in Tijuana is nontoxic, natural, or less invasive. Campaigns with this rationale could point to the perceived toxicity of conventional care, particularly chemotherapy, and suggest that CAM care in Tijuana used gentler and more natural modalities. (5) Treatment in Tijuana offers the newest or most advanced technology and treatment types. These campaigns could include statements that the regulatory system in the recipient’s home country was too restrictive and made these treatments unavailable domestically, discussion of perceived “cutting edge” technologies, or appeals to the training and credentials of staff at the preferred provider. We then applied these codes to each campaign, allowing for multiple rationale codes per campaign. All of these codes were independently confirmed by 2 authors. Discrepancies were discussed among the first 3 authors and coding was refined until consensus was reached.

**Figure 1:**
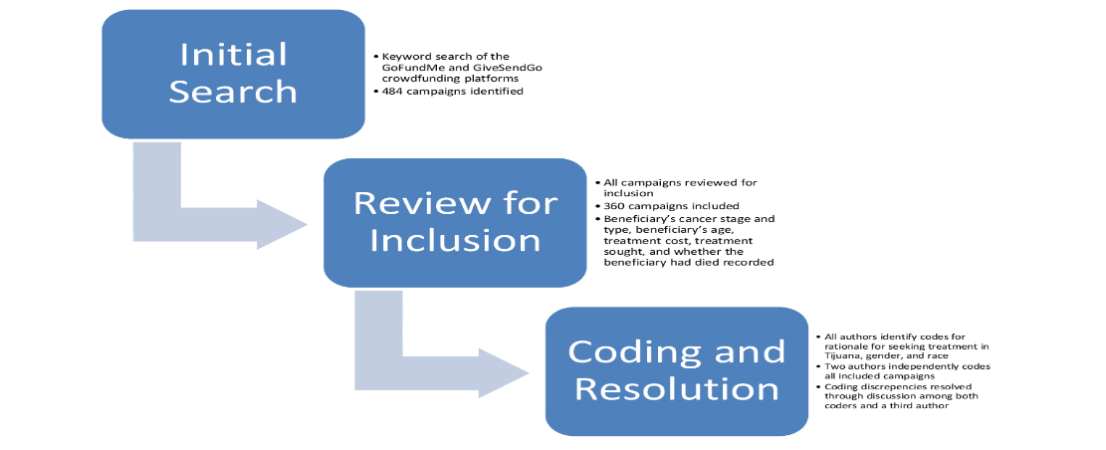
Workflow.

We reviewed all campaigns and assigned a gender and racial category to each beneficiary. Gender categories included women, men, nonbinary, and undetermined. Gender identification was based on campaign photos, pronoun usage in the campaign text, and other textual cues. Racial categories were identified in consultation with publications on racial characteristics in crowdfunding in order to identify commonly used categories within this area of scholarship [17-20] and further refined through discussion among the reviewers. These categories were then assigned based on campaign images, the beneficiary’s and family members’ names, non–English language campaign text, and other textual cues as informed by the reviewers’ experience and prior publications using racial data in crowdfunding campaigns. Two authors each independently assigned a gender and racial category for each campaign or noted uncertainty regarding categorization. Discrepancies in these codes were discussed among each reviewer and a third author, and they were resolved where possible after exchanging rationales for assigning discrepant codes. Where consensus was not achieved or there was not enough information in the campaign to decide, the relevant category was assigned as undetermined. While challenging, collecting and analyzing gender and racial data can yield important insight around medical access and health equity (see Figure 1) [21].

### Ethical Considerations

Ethics approval was not required for this study as per the Tri Council Policy Statement (TCPS2; Article 2.2) [22], as all data were posted in the public domain and the individuals to whom the information refers have no reasonable expectation of privacy.

## Results

This process identified 360 crowdfunding campaigns (GoFundMe, n=311; GiveSendGo, n=49). These campaigns raised US $5,275,268.37 (median US $7685) with a range of US $0-$220,812. They requested US $17,032,458.06 (median US $45,000) with a range of US $0-$250,000. Contributions were received from 38,212 (median 46) donations with a range of 0-1495. In total, 352 (97.8%) of the campaigns received some funding and 22 (6.1%) reached or exceeded their fundraising goals. These campaigns were shared online 86,907 (median 124) times on social media with a range of 0-2600 shares. Table 1 presents these data by crowdfunding platform, and Table 2 presents this information by quartile.

**Table 1:** Fundraising by crowdfunding platform.

	US $ raised	Median raised	US $ requested	Median requested	Total donations, n	Median donations	Total shares, n	Median shares	Receiving funding, %	Meeting funding goal, %
GiveSendGo	809,428.50	8250.00	2,326,394.86	45,000	2607	32	1229	15	83.7	14.3
GoFundMe	4,465,839.87	7525	14,706,063.20	45,000	35,605	49	85,678	150	100	4.8
Both platforms	5,275,268.37	7685	17,032,458.06	45,000	38,212	46	86,907	124	97.8	6.1

**Table 2:** Fundraising by quartile.

	First quartile	Second quartile	Third quartile	Fourth quartile
Funding received (US $)	0-2620	2640-7605	7765-18,750	18,895-220,812
Funding requested (US $)	0-25,689.80	25,900-45,000	45,000-55,000	55,867-250,000
Donations, n	0-18	19-50	51-105	107-1495
Shares, n	0-23	23-128	130-331	332-2600

### Beneficiary and Provider Characteristics

In total, 251 (69.7%) campaign beneficiaries were women and 109 (30.3%) were men (no beneficiaries identified as nonbinary). The GiveSendGo platform skewed more heavily toward women (n=39, 79.6%) compared to the GoFundMe platform (n=212, 68.2%). The beneficiary’s age was identified in 105 campaigns. Ages ranged from 18 to 71 years with a median age of 44 years. Most beneficiaries were White (n=256, 71.1%) followed by Latino (n=39, 10.8%), Black (n=38, 10.6%), East Asian (n=10, 3.1%), Middle Eastern and South Asian (n=5, 1.4%), and Indigenous (n=2, 0.6%) beneficiaries with 7 (2.5%) campaigners not identified (see Table 3). White and women beneficiaries outraised other groups (see Table 4). Campaigners were most commonly located in the United States (85%), followed by Canada (6.4%) and the United Kingdom (3.6%). Over 75% of campaigns sought treatment at 3 clinics: Hope4Cancer (n=146, 40.6%), Centro Hospitalario Internacional Pacifico (CHIPSA; n=81, 22.5%), and Oasis of Hope (n=44, 12.2%; see Table 5).

**Table 3:** Beneficiary race.

Race	GoFundMe, n (%)	GiveSendGo, n (%)	Total, n (%)
White	211 (67.8)	45 (91.8)	256 (71.1)
Latino	38 (12.2)	1 (2.0)	39 (10.8)
Black	38 (12.2)	0 (0.0)	38 (10.6)
East Asian	10 (3.2)	1 (2.0)	11 (3.1)
Middle Eastern and South Asian	5 (1.6)	0 (0.0)	5 (1.4)
Indigenous	2 (0.6)	0 (0.0)	2 (0.6)
Uncertain	7 (2.3)	2 (4.1)	9 (2.5)

**Table 4:** Outcomes by gender and race.

	Median donations	Median raised (US $)	Median requested (US $)
Women	49.5	7959	45,000
Men	41	7085	45,000
White	50	8516	47,770.56
Latino	41	4060	30,000
Black	48	4439	45,000
East Asian	44	7144.21	40,000
Middle Eastern or South Asian	370	25,357.30	44,933.80
Indigenous	104.5	20,051	37,500
Uncertain	17	3610	37,500

**Table 5:** Intended provider.

Clinic Name	Value, n (%)
Hope4Cancer	146 (40.6)
CHIPSA^a^	81 (22.5)
Oasis of Hope	44 (12.2)
ITC^b^	24 (6.7)
Sanoviv	11 (3.1)
Immunotherapy Institute	6 (1.7)
Gerson Institute	5 (1.4)
Hoxsey	4 (1.1)
Advanced Gerson	3 (0.8)
Health Institute de Tijuana	2 (0.6)
Integrative Cancer Centers of America	1 (0.3)
Medgate Baja	1 (0.3)
Northern Baja Gerson Center	1 (0.3)
Stella Maris Clinic	1 (0.3)
Unidentified	30 (8.3)

^a^CHIPSA: Centro Hospitalario Internacional Pacifico.

^b^ITC: Immunity Therapy Center.

A total of 125 campaigners stated the cost of the treatments they sought in Tijuana, which ranged from US $11,000 to US $100,000 (median US $45,000). The most common cancer types or locations disclosed in these campaigns were breast (26.9%), colorectal (14.2%), and pancreatic (7.2%) cancers. Of the 205 campaigns that stated the beneficiary’s cancer stage, these skewed toward later stages with 161 (78.6%) at stage 4 followed by 35 (17.1%) stage 3, 6 (2.9%) stage 2, and 3 (1.5%) stage 1. A total of 67 (18.6%) beneficiaries were identified as having died after the start of the campaign. Common treatments sought included immunotherapy (n=94), dietary supplements (n=44), detoxification (n=36), Gerson therapy (n=31), ozone and oxygenation therapies (n=32), hyperbaric oxygen therapy (n=27), hyperthermia (n=26), vitamin C (n=26), dendritic cell therapy (n=20), light-based (infrared, laser, photodynamic) treatments (n=16), vitamin B17 (n=14), low dose chemotherapy (n=12), sono-photodynamic therapy (n=10), Coley’s therapy (n=8), insulin potentiation therapy (n=8), cryotherapy (n=7), curcumin (n=7), and pulsed electromagnetic therapy (n=7).

### Rationales for Seeking Treatment in Tijuana

These campaigns offered a variety of rationales for seeking alternative cancer treatment in Tijuana, including multiple rationales in the same campaign. The most common rationale (30.9%) was that this treatment was perceived as offering the greatest possible efficacy in terms of curing the beneficiary’s cancer or extending their lifespan. Campaigns included both general claims about the success of treatments at the facility and highly specific numbers such as “a high success rate of over 90 percent.” These claims were bolstered by the providers (“they claim that they can both stop the cancer he has”) and patient testimonials (“success stories at this centre have been incredible to read”).

The second most common rationale (23.6%) for seeking treatment in Tijuana was that the care offered domestically was not curative and so they desired to continue seeking curative treatment. These campaigns frequently described an experience where domestic practitioners stated a low survival rate or duration for the beneficiary and suggested they explore hospice or palliative care. Accepting a lack of curative treatment options was often rejected, positioning the recipient as a “fighter” who does not “give up as easily” or explaining that they had others who needed them to survive (“I NEED to be here to see my wee babies grow up and to be their mummy”).

Beneficiaries also sought treatment in Mexico (19.3%) because these facilities were seen as treating the whole person in a caring way. These campaigns often referred to the alternative care they sought as “holistic” or targeting the “whole person” including their “mind, spirit and emotions.” Others described the caring approach of the clinic staff and personalized nature of their care: “the doctors are so kind, warm, attentive and the treatment plan is truly individualized!”. Some of these campaigns particularly flagged the spiritual or religious dimension of the providers in Tijuana, including describing one clinic as “run by doctors and staff that are all Spirit-filled believers.”

These clinics were also seen as offering less toxic and less invasive, natural treatment options (17.6%). Campaigners sought “non-toxic cancer therapies” that “target only cancer cells,” leaving the rest of the person intact. Chemotherapy and radiation treatments were seen as “harsh” based on the beneficiary’s past experience or witnessing the treatment of loved ones. Instead, the “natural” treatments offered in Tijuana could particularly “rebuild” the recipient’s immune system. Other campaigners objected to surgery, including the removal of reproductive organs or other life-altering changes. Alternative care offered “less invasive treatments” that avoided these outcomes.

Least commonly (8.6%), these campaigns were motivated by the perceived technological superiority of the treatments offered in Tijuana. These treatments were frequently described as being “experimental,” “cutting edge,” or “state of the art.” Specific clinics were flagged as having a reputation as global leaders in “advanced” cancer treatments or being “known for being on the forefront” of cancer care. In some cases, campaigners noted that the clinics were free from regulatory limits on new treatments domestically, and therefore, “able to do certain treatments that are not FDA approved here in the US” (See Table 6).

**Table 6:** Alternative treatment rationales.

Rationale	GoFundMe, n (%)	GiveSendGo, n (%)	Total, n (%)
Efficacy	166 (30.6)	29 (32.6)	195 (30.9)
Domestic not curative	137 (25.3)	12 (13.5)	149 (23.6)
Whole person	98 (18.1)	24 (27.0)	122 (19.3)
Natural	94 (17.3)	17 (19.1)	111 (17.6)
Newest technology	47 (8.7)	7 (7.9)	54 (8.6)

## Discussion

The findings from this study affirm earlier studies of crowdfunding for CAM cancer treatment. Crowdfunding beneficiaries seeking CAM cancer treatments in this study most commonly experienced breast and colorectal cancers, as has been seen in previous studies [10,23,24]. The beneficiaries in this study were most commonly women (69.7%). This closely matches previous findings of 64.3% and 70% of beneficiaries as female [10,24]. As with the previous studies, most beneficiaries were described as having stage 4 cancer [6,10].

Campaigns in this study generally had higher median fundraising goals (US $45,000) and donations (US $7685) than in previous studies. Holler et al found that crowdfunding campaigns for US beneficiaries for conventional and alternative treatments raised median US $1610 of US $9000 requested and Song et al [10] found of a median goal of US $15,000 for CAM cancer treatments with US $2870 raised [10,23]. Another study that included non-US GoFundMe campaigns found median US $19,880 requested and US $5055.50 raised [9]. Only one study of US-based campaigns for alternative treatment abroad found higher median donations. These campaigns raised median US $7833 of US $35,000 requested [6]. As the campaigners generally lived in the United States, Canada, and the United Kingdom, travel costs likely influenced higher fundraising goals, which may have in turn encouraged more giving. Moreover, the median stated direct cost of treatment (US $45,000) suggests that treatment costs in the providers included in this study were higher than those in previous studies.

Previous studies of crowdfunding for CAM cancer treatments have not examined racial characteristics of campaign beneficiaries. Among a sample of 2618 US-based medical campaigns on the GoFundMe platform, White beneficiaries were found to be most common (73.7%), followed by Latino (12.3%) and Black (9.4%) beneficiaries [20]. This and other studies have also shown that White campaigners tend to raise more money than Black beneficiaries [17,18]. Our study findings are consistent with these observations.

These campaigns conferred many benefits to clinics in Tijuana. Most directly, these campaigns supplied these clinics with a potential new source of revenue. Not all of the over US $5 million raised through these campaigns will go to these clinics as it was also used for indirect costs like travel and time off work and some beneficiaries were unable to travel to Tijuana for treatment. Nonetheless, the funding raised through these campaigns supplemented campaigners’ insurance, savings, and other financial resources and, as a result, these clinics may have received more paying clients. Moreover, these campaigns serve as highly effective advertising about the clinics’ perceived merits to people viewing and contributing to them. Campaign claims about the efficacy of these clinics and their superiority to domestic providers are presented in the form of patient testimonials; as such, they are likely to be highly effective forms of advertisements, reaching a wide audience via social media. In this way, crowdfunding campaigns create a positive feedback loop for targeted clinics where customers using crowdfunding generate highly positive social media about the clinics, which in turn generates new potential customers, some of whom likely turn to crowdfunding themselves.

While these campaigns raised a great deal of money collectively, they typically fell short of their individual fundraising goals; the median US $7685 raised was well short of the median US $45,000 requested and only 6.1% of the campaigns reached or exceeded their fundraising goals. These fundraising goals were likely driven by the substantial cost of treatment in Tijuana as the campaigns indicated a median treatment cost of US $45,000. Many campaigners were unable to afford their desired treatments and others likely drained savings or went into debt to do so. This issue was particularly acute for Black and Latino beneficiaries who raised median US $4439 and US $4060, respectively, compared to US $8516 for White beneficiaries. Outside of these financial implications, these campaigns entail the loss of privacy for campaign beneficiaries and their families through public exposure of their medical, financial, and other details. Other campaigners for cancer care have described feeling uncomfortable or humiliated from having to ask others for financial support for their care [25].

Previous studies have flagged the use of markers of legitimacy among providers of alternative medical treatments. These markers include scientific and research-based language that helps build trust in potential clients [9]. The rationales for seeking CAM treatment in these campaigns tended to emphasize the efficacy of the interventions they sought and, to a lesser extent, how it was cutting-edge technology and not available domestically. By comparison, campaigns emphasizing the natural dimensions of the treatment or caring and spiritual nature of the facility’s staff were less common. Specific treatments like immunotherapy borrow from language used in more conventional and evidence-based treatments such as Chimeric Antigen Receptor T-cell therapy. Thus, patients may be unclear about the actual nature of and evidence for the treatments they seek abroad [26]. This may mark a divergence from previous studies that have found interest in combatting cancer through immune boosting modalities that focus primarily on “natural” products to do so [24].

Campaigns on both the GoFundMe and GiveSendGo crowdfunding platforms had the same median fundraising goal. While campaigns on GoFundMe had a larger median number of donations and shares, GiveSendGo campaigns had a larger median amount raised and relatively more campaigns reach their goal. These differences could be due to the higher ratio of White beneficiaries using GiveSendGo or other factors that lead to higher amounts given per donor on that platform. GiveSendGo campaigns put relative emphasis on caring for the whole person, including the spiritual dimensions of care, as a motivation for seeking treatment in Tijuana. These differences display how the populations using crowdfunding platforms can differ despite seeking the same treatments in the same location. Additional study of these differences is needed, particularly given the growth of the GiveSendGo.

This study had several limitations. Coding for the beneficiary’s gender and race typically relies on the perceptions of the coders and may be inaccurate. Some campaigns may have been removed prior to data collection, particularly campaigns from earlier in the inclusion period. Campaigns that met our inclusion criteria but did not mention a clinic name or specify seeking alternative cancer treatment in Tijuana or Baja, Mexico, would not have been identified. Additionally, some campaigns may have continued to raise money after the end of data collection. Thus, this study likely understates the number and fundraising total of campaigns for CAM cancer treatment in Tijuana, Mexico.

As has been previously established, crowdfunding is actively used to raise money to access CAM cancer treatments. These campaigns spread problematic misinformation about the likely efficacy of these treatments, funnel money and endorsements to these clinics, and leave many campaigners short of the money needed to pay for them while costing beneficiaries and their loved ones time, privacy, and dignity. This study affirms that Tijuana, Mexico, is a popular destination for these campaigners and that this interest persists following the COVID-19 pandemic. While most of these campaigns fell well short of their goals, Black and Latino beneficiaries were particularly unsuccessful. This study also demonstrates an evolving landscape of CAM cancer treatments generally, and in Tijuana specifically, with increased marketing of immunotherapy as a form of treatment. This study demonstrates both the value of close examination of specific destinations for CAM cancer treatments and for how distinct populations may be drawn to different crowdfunding platforms.

## Abbreviations

CAM: complementary and alternative
CHIPSA: Centro Hospitalario Internacional Pacifico

## Data Availability

The data are available on request from the corresponding author.
